# P-2151. Interactive Dashboard for Tracking Malaria across the Veterans Health Administration (VA), 2000 – 2023

**DOI:** 10.1093/ofid/ofae631.2305

**Published:** 2025-01-29

**Authors:** Lauren Epstein, Ariana Paredes-Vincent, Christopher Ogston, Maria C Rodriguez-Barradas, Robert A Bonomo, Sheldon T Brown, Christopher Woods

**Affiliations:** Atlanta VA, Atlanta, Georgia; Veterans Health Administration, New York, New York; Veterans Health Administration, New York, New York; Michael E. DeBakey VAMC and Baylor College of Medicine, Houston, Texas; Case Western Reserve University/ Louis Stokes Cleveland VA Medical Center, Cleveland, OH; James J Peters VAMC, Bronx, NY; Durham VA Medical Center/DUke, Durham, North Carolina

## Abstract

**Background:**

The VA utilizes a comprehensive data system that is updated in real time and can be used to track trends and geographic shifts in emerging diseases across the U.S. Using malaria as an example, our goal was to illustrate the (1) feasibility of creating a dashboard to conduct public health surveillance using VA data, (2) assess trends, and (3) compare the VA and national trends using publicly available data.

**Figure 1:** Incidence of malaria across the VA System by location of diagnosis (inpatient vs. outpatient), 2000 – 2023.

**Figure:**
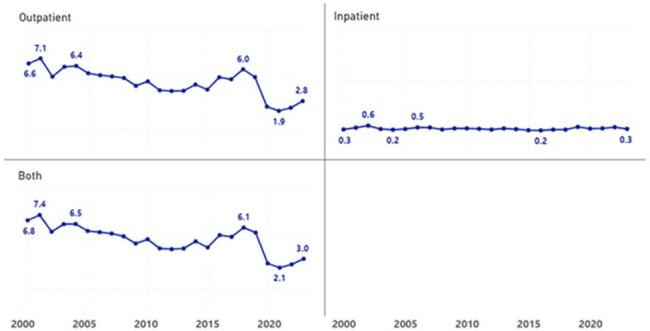

Rate per 100,000 VA patients.

**Methods:**

This project was developed as part of VA SHIELD, a comprehensive biorepository of specimens and clinical data that started in 2020 to address national health threats across the VA system. Within this overarching objective, we assessed trends in malaria cases across all VA facilities from 2000 – 2023 using the VA’s Corporate Data Warehouse.

We identified unique cases using 13 ICD-9 and 13 ICD-10 codes for malaria. We calculated incidence rates (per 100,000 population) based on all patients that received care at any type of VA facility (inpatient, outpatient). We compared the yearly VA rates with national rates using publicly available data in CDC’s WONDER database.

**Figure 2:** Comparison of malaria trends, VA vs. national surveillance (2000 – 2023)

**Figure:**
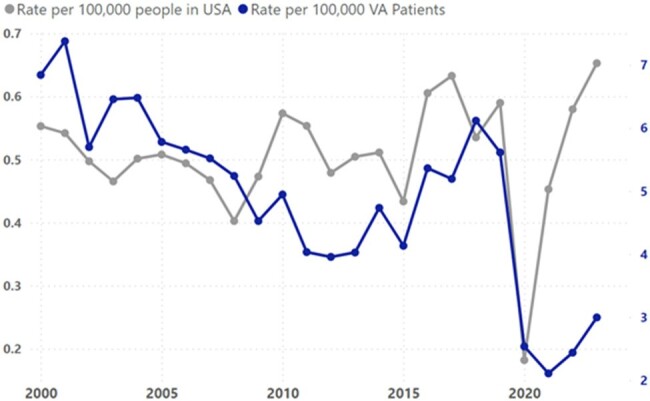

VA rates includes both inpatient and outpatient locations combined.

**Results:**

We identified 6,415 cases of malaria, ranging between 141 (2021) and 387 (2018) annually. The annual rate of malaria varied between 2.11 (2021) and 7.38 (2001) cases per 100,000 VA patients. The annual rate of malaria diagnosed among hospitalized patients ranged between 0.18 (2016) and 0.64 (2002) cases per 100,000 VA patients. In comparison, CDC reported national malaria rates varied between 0.18 (2020) and 0.65 (2023) per 100,000 persons. In 2020, there was a decrease in malaria rates that was observed both nationally and among the VA population.

**Conclusion:**

Using the diagnosis of malaria, we show how the VA’s vast data system can contribute to public health surveillance. Historically, locally acquired malaria infections in the U.S. are rare. However, in 2023, 10 locally acquired cases of malaria were reported from 3 states, illustrating the possibility for wider transmission. This project illustrates the potential of the VA to detect rare infections in real time and possibly serve as a national sentinel surveillance platform. Future endeavors include prospective surveillance with specimen and clinical data collection in order to augment malaria public health surveillance.

**Disclosures:**

All Authors: No reported disclosures

